# Disjunction and Vicariance Between East and West Asia: A Case Study on *Euonymus* sect. *Uniloculares* Based on Plastid Genome Analysis

**DOI:** 10.3389/fpls.2022.825209

**Published:** 2022-03-11

**Authors:** Shayan Jamshed, Joo-Hwan Kim

**Affiliations:** Department of Life Sciences, Gachon University, Seongnam, South Korea

**Keywords:** chloroplast genome, divergence, dispersal, disjunction, East Asia, *E.* sect. *Uniloculares*, vicariance, West Asia

## Abstract

Scientists have long been captivated by biogeographic disjunctions, and disjunctions between East Asia and North America have been particularly well-studied at the genus and family levels. By contrast, disjunctions between eastern and western Asia have received less attention. *Euonymus* L. is taxonomically divided into two sections based on the number of cells in anthers as follows: *E*. sect. *Uniloculares* has one-celled anthers and occurs mainly in Asia, whereas *E*. sect. *Biloculares* has two-celled anthers and is distributed globally. We used Illumina sequencing to investigate the genomes of four species in sect. *Uniloculares*. The chloroplast (cp) genomes are highly conserved (157,290–158,094 bp). Pseudogenisation of *ndh*F and intron loss in *rps*16 was detected. Based on the cp genomes of the four species of *E*. sect. *Uniloculares*, we propose a novel hypothesis of disjunction between eastern and western Asia. Biogeographic reconstruction and molecular dating revealed that sect. *Uniloculares* separated from its sect. *Biloculares* forebears 4.0 Mya during the Pliocene era. The radial diversification of sect. *Uniloculares* from East Asia and the establishment of the western Asian clade during the Pleistocene era (1.9 Mya) were the results of both dispersal and vicariance, making the section the youngest diverged clade conforming to age estimation. The centre of origin of sect. *Uniloculares* was determined to be in East Asia. Disjunctions and diversification between eastern and western Asia in sect. *Uniloculares* are thought to have been caused by changes in monsoon patterns, temperature variations, and the emergence of the Gobi Desert.

## Introduction

Understanding intercontinental disjunction patterns is among the main objectives of biogeography (Cox et al., 2016; Costa et al., 2020; Namgung et al., 2021). Such disjunctions and fragmentations have resulted from continental drift, the formation of the Bering land bridge, the formation of deserts and lakes, and global climatic oscillations during the Cenozoic era (Ickert-Bond and Wen, 2006; Liao et al., 2007; Nie et al., 2008; Kim et al., 2015; Kim et al., 2017). Disjunctions between the temperate regions of eastern North America and East Asia (Wen, 1999; Xiang et al., 2000; Wen, 2001), western North America and southern Europe (Wen and Ickert-Bond, 2009; Mao et al., 2010), and eastern and western North America (Xiang et al., 1998) have received much attention (Xiang et al., 1998; Wen, 1999; Deng et al., 2015; Kim et al., 2017; Song et al., 2020). However, less emphasis has been placed on understanding the disjunction between East and West Asia (Song et al., 2020).

*Euonymus* L. is among the largest genera in the Celastraceae, comprising 129–200 species (Leonova, 1974; Ma, 2001; Savinov and Baikov, 2007; Gavrilova et al., 2018) of deciduous and evergreen shrubs and small trees (Gavrilova et al., 2018). The genus is widely distributed throughout tropical and temperate regions (Blakelock, 1951; Ma, 2001; Xia et al., 2018), but it is concentrated within east, south, and southeast Asia and the Himalayas (Ma, 2001; Yao et al., 2018). Blakelock (1951) also noted its occurrence in Madagascar, North Africa, and Australia and concluded that *Euonymus* is a cosmopolitan genus (Blakelock, 1951).

With respect to classification, Ma (2001) divided *Euonymus* into five sections, such as sects. *Uniloculares*, *Echinococcus*, *Illicifolia*, *Melanocarya*, and *Euonymus*, based on capsule texture (angular, echinate, or smooth). In contrast, Rouy and Foucaud, in Flora of France (Fl. De France 4, 158-9, 1897), recognised the following two sections: the *Biloculares*, with bilocular anthers (e.g., *Euonymus europeaus*), and the *Uniloculares*, with unilocular anthers (e.g., *Euonymus latifolius*) (Blakelock, 1951).

Species in *E.* sect. *Uniloculares* are described as deciduous, evergreen shrubs, or small trees and are characterised by tetra- or pentamerous flowers (Ma and Funston, 2008), winged capsules, and one-celled anthers. Sect. *Uniloculares* has been alternatively classified as subgenus *Kalonymus*; however, the key characteristics of subgenus *Kalonymus* (one-celled anthers and winged capsules) are similar to those of sect. *Uniloculares*, and as such subgenus *Kalonymus* was declared illegitimate (McNeill et al., 2012; Du et al., 2016). Of the 16 species discussed in this section, only *E. latifolius* occurs in western Asia, Europe, and North Africa. The other 15 species, including *Euonymus macropterus*, Euonymus *sachalinensis*, and Euonymus *oxyphyllus*, are distributed throughout East Asia and the Russian Far East. China is a hotspot for species discussed in this section and hosts eight endemic species, including *Euonymus schensianus* and *Euonymus szechuanensis* (included in this study) (Ma and Funston, 2008).

We used the members of sect. *Uniloculares* as a model to understand vicariance and disjunction, as the section exhibits a disjunction between eastern and western Asia. We used the whole chloroplast (cp) genomes of six species in sect. *Uniloculares*, of which we sequenced the following four species: *E. macropterus*, *E. sachalinensis*, *E. oxyphyllus*, and *E. latifolius*. The genomes of *E. schensianus* and *E. szechuanensis* were obtained from the National Centre for Biotechnology Information (NCBI) GenBank. *E. macropterus*, *E. sachalinensis*, and *E. oxyphyllus* are distributed in East Asia and the Russian Far East, whereas *E. latifolius* is distributed in western Asia, Europe, and North Africa, making this section a suitable model for exploring disjunction and evolution based on the whole cp genome.

Analysis of cp genomes is widely used in phylogenetic studies. In angiosperms, the cp genome is transferred from the female parent (Palmer et al., 1988). It is a type of plastid with a double-layered membrane and thylakoid structures with a high concentration of chlorophyll, which plays a vital role in photosynthesis and other biochemical processes (Neuhaus and Emes, 2000; Cheng et al., 2020; Liang et al., 2020). Similar to mitochondria, cp is among the organelles that have their genome (Cheng et al., 2020). Gene deletions, duplications, mutations, and rearrangements have been observed in the cp genomes of angiosperms (Lee et al., 2007). Phylogenetic studies use cp genomes as they are more highly conserved among taxa than nuclear and mitochondrial genomes. Genomes may provide markers for phylogenetic analyses and may be used to estimate the time of divergence of higher taxonomic ranks (Moore et al., 2010; Lee et al., 2019).

The insights obtained from phylogenetic, age estimation, and biogeographic studies rely on analysis of the whole cp genome, which is considered essential to infer backbone phylogeny and phylogenetic trees. Analysis of the cp genome may also help to resolve the relationships among complex groups of plants, which remain unclear due to taxonomic gaps.

## Materials and Methods

### Taxon Sampling and DNA Extraction

Samples of *E. macropterus*, *E. oxyphyllus*, and *E. sachalinensis* were collected from different sites in South Korea, whereas *E. latifolius* samples were collected from the Tsytsin Main Botanical Garden, Russian Academy of Sciences (RAS), Moscow, Russia (refer to Table 1). All samples were dried using silica gel, and voucher specimens for the Korean samples were prepared and submitted to the Gachon University Herbarium (GCU). Dried samples were used for DNA extraction using the common 2 × cetyltrimethylammonium bromide (CTAB) method (Doyle and Doyle, 1987). Concentrations of extracted DNA were measured using a spectrophotometer (BioSpec-nano Shimadzu, Kyoto, Japan). The DNA bands were observed using gel electrophoresis, and samples with good-quality bands were subjected to quality tests.

**TABLE 1:** *Euonymus* sect. *Uniloculares* samples’ collection sites.

S.no	Taxon	Collected country/region	Botanical garden/arboretum/ field/reference
1	*Euonymus latifolius* (L.) Mill.	Caucas	Tsytsin Main Botanical Garden RAS, Moscow, Russia
2	*Euonymus macropterus* Rupr.	South Korea	Sangwonsa temple to Jeokmyeol bogung, Odaejan Mt. Gangwan-do
3	*Euonymus oxyphyllus* Miq.	South Korea	Korean National Arboretum
4	*Euonymus sachalinensis* (F. Schmidth) Maxim.	South Korea	Korean National Arboretum
5	*Euonymus szechuanensis* C.H Wang	Sichuan Province, China	Ziyang County (Wang et al., 2020)
6	*Euonymus schensianus* Maxim	Shaanxi Province, China	Arboretum of Northwest A&F University (Wang et al., 2017)

### Chloroplast Genome Assembly and Annotation

DNA samples with concentrations of >20 ng/μl were transferred to the next-generation sequencing using the Illumina MiSeq sequencing system (Illumina, Seoul, South Korea). Sequencing libraries were prepared using an Illumina TruSeq Nano DNA Library Preparation Kit. Raw reads (300-bp paired-end reads) were trimmed to remove regions with error probabilities > 3% per base using Geneious version 7.1.9 (Kearse et al., 2012). Total reads, assembled reads, and coverage are summarised in Supplementary Table 5. Paired-end reads were assembled by performing a “map to reference” using the cp genome of *E. schensianus* (GenBank Acc. no: KY511610), which is accessible in NCBI. Gene content was annotated in Geneious version 7.1.9 using *E. schensianus* as a reference and applying a 75% similarity index to identify genes. Following the generation of quadripartite structures, further steps were carried out. The start and end of transfer RNAs (tRNAs) were confirmed using tRNAScan with the default settings (Chan and Lowe, 2019). Circular and linear cp maps were then converted into graphical maps using OrgaellarGenomeDRAW (Greiner et al., 2019).

### Comparative Genomic Analysis

We compared the cp genomes of six species in sect. *Unilocualres* using the Shuffle-LGAN mode in mVISTA (Frazer et al., 2004). *Euonymus hamiltonianus* (KY926695) was used as a reference genome. To evaluate differences among the six species, whole cp genomes, including coding genes, tRNAs, ribosomal RNAs (rRNAs), and intergenic spacers, were aligned using MAFFT version 7.017 embedded in Geneious version 7.1.9 (Katoh et al., 2002). Nucleotide diversity (π) was calculated using DnaSP version 6.12.03 (Rozas, 2009). The structure and junctions between inverted repeats (IRs), large single copies (LSCs), and small single copies (SSCs) were investigated using IRscope (Amiryousefi et al., 2018).

### Phylogenetic Analysis

A total of 11 *Euonymus* species were used to construct a phylogenetic tree; four were sequenced as part of this study, whereas the data for the remaining seven species were obtained from NCBI GenBank (Table 2 and Supplementary Table 1). We extracted and aligned 80 protein-coding genes using MAFFT version 7.017 embedded in Geneious version 7.1.9 (Katoh et al., 2002). Maximum parsimony (MP), maximum likelihood (ML), and Bayesian inference (BI) were used to infer relationships among the 11 *Euonymus* L. species, while six other genera in the Celastraceae were used as outgroups. Maximum parsimony was conducted using PAUP version 4.0 (Wilgenbusch and Swofford, 2003), using equal character weighting and treating gaps as missing data. Searches of 1,000 random additions used tree bisection and reconnection branch swapping, and MulTree permitted 10 trees to be held at each step. Bootstrap analysis, with the same parameters, was used to find internal support. J-Model test version2.1.10 was used to identify the best model based on Akaike’s Information Criterion (AIC). AIC values indicated that TVM + I + G was the best model, and we used this model for the ML and BI analyses. IQ-TREE was also used for the ML analysis (Trifinopoulos et al., 2016). A support value was calculated with 1,000 ultrafast bootstrap replicates. We used MrBayes version 3.2.6 for the BI analysis (Ronquist et al., 2012). Two runs were conducted from random trees for a minimum of 1,000,000 generations. One tree was sampled every 1,000 generations. The first 25% of trees were discarded as burn-in, and the remaining trees were used to construct a 50% majority-rule consensus tree, with the proportion of bifurcations in the consensus tree provided as a posterior probability (PP) to estimate the robustness of half of the BI tree. Effective sample size (ESS) values were then examined for model parameters (≥200). Trees were edited using FigTree version 1.4.4 (Rambaut, 2020).

**TABLE 2:** Chloroplast genome features of *Euonymus* sect. *Uniloculares* species.

Name	NCBI accession number	Genes/ pseudogenes	rRNA	tRNA	LSC	SSC	IRa	IRb	Total base pairs	GC%	AT%
*E. oxyphyllus* Miq.	OL770078	134/3	8	37	85,924	18,543	26,697	26,697	157,861	37.1	62.9
*E. macropterus* Rupr.	OL770077	136/3	8	39	85,499	18,460	26,709	26,709	157,377	37.2	62.8
*E. latifolius* (L.) Mill.	OL770076	135/3	8	38	86,161	18,581	26,676	26,676	158,094	37.1	62.9
*E. sachalinensis* (F. Schmidth) Maxim.	OL770079	134/3	8	37	85,404	18,476	26,705	26,705	157,290	37.2	62.8
*E. schensianus* Maxim.	KY511610	133/3	8	37	86,026	18,528	26,574	26,574	157,702	37.2	62.8
*E. szechuanensis* C.H Wang	MH853828	134/3	8	37	86,401	18,472	26,296	26,296	157,465	37.2	62.8

### Divergence Time Estimation and Fossil Constraints

Fossil data for *Maytenus* spp. (17.4–7.1 Mya), *Salacia* spp. (7.1–2.8 Mya), and *Celastrus madagascariensis* (21.7–11.7 Mya), all from Africa and Madagascar, were used as minimum age constraints for three nodes (Bacon et al., 2016). To infer time of divergence, we used the uncorrelated relaxed molecular clock model for Markov Chain Monte Carlo (MCMC) simulation in BEAST version 1.8.3, featured in Beauti version 1.8.3. The Yule speciation tree prior (Gernhard, 2008) was used with an uncorrelated relaxed molecular clock. The stem nodes C1–C3 were calibrated using a normal distribution because of the availability of means for the fossil data used in age estimation (Figure 7). C1 and C3 had mean values of 12.2 and 16.5 (SD = 2.9), respectively, whereas C2 had a mean value of 4.7 (SD = 1.2). MCMC length was limited to 100 million generations, and parameter sampling was conducted every 1,000 generations. Tracer version 1.65 was used for convergence (Rambaut and Drummond, 2003), and ESS was assessed. A maximum clade credibility tree was generated using TreeAnnotator version 1.8.3, following the removal of the first 25% of trees as burn-in and to display the mean age and 95% of highest posterior density (HPD) interval for each node (Drummond and Rambaut, 2007). The tree was annotated using FigTree version 1.4.4 (Rambaut, 2020).

### Biogeographic Analyses

Eight areas were defined within the geographic ranges of the 11 focal species (five species from sect. *Biloculares* and six species from sect. *Uniloculares*) based on available literature (Agea et al., 2021) and web sources, particularly Plants of the World Online (POWO, 2019). These areas included East Asia; the Russian Far East; western Asia; south, southeast, and central Europe; North Africa; south and southeast Asia and the eastern and western Himalayas; North America; and South Africa. Ancestral area reconstruction was conducted using Reconstruct Ancestral State in Phylogenies (RASP) version 2.1b (formerly S-DIVA) (Yu et al., 2010; Yu et al., 2013).

## Results

### Comparative Plastome Analysis

**FIGURE 1:**
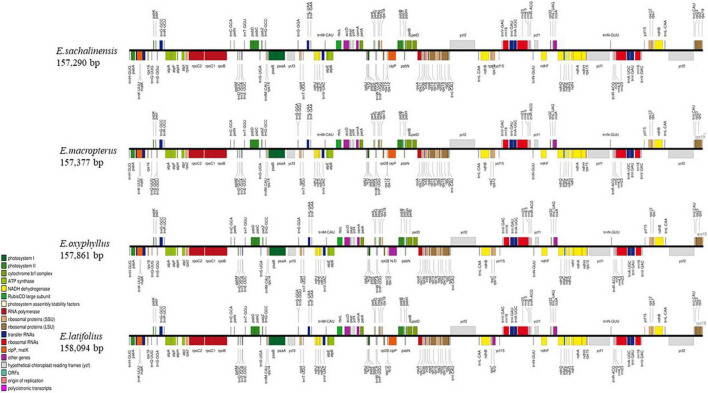
Linear gene map of four species of *Euonymus* sect. *Uniloculares*. Genes shown above are transcribed clockwise, and genes shown below are transcribed counterclockwise. The functional group of genes is shown by the colour code on the left.

The whole cp genomes of four species of sect. *Uniloculares*, including *E. latifolius*, *E. sachalinensis*, *E. macropterus*, and *E. oxyphyllus*, were constructed. The results indicated that all four species had typical cp genomes consisting of LSC and SSC regions and two IR copies, which are typically referred to as IRa and IRb. The genomes of two additional species in sect. *Uniloculares* (*E. schensianus* and *E. szechuanensis*) were obtained from GenBank. The four newly assembled cp genomes were compared with those of the aforementioned species. No discernible differences were observed among the six species. However, *E. latifolius* had a large genome (158,094 bp), whereas that of *E. sachalinensis* was small relative to the other species (157,290 bp). The LSC in *E. oxyphyllus* had more base pairs (85,924 bp). *E. latifolius* also had a large number of base pairs in the SSC (18,581 bp), whereas the IR in *E. macropterus* comprised 26,709 bp (Figure 1).

**FIGURE 2:**
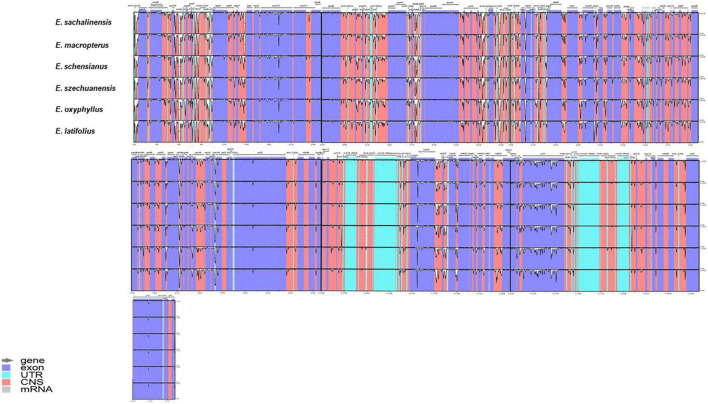
Using mVISTA, six chloroplast genomes of *Euonymus* sect. *Uniloculares* were aligned in parallel using *Euonymus hamiltonianus* as a reference. The transcriptional directional order of genes is indicated by arrows above. The identity on *Y*-scale axis ranges from 50 to 100%. Colour-coded regions include exon, intron, and non-coding regions.

The numbers of coding genes and rRNAs were similar among species. *E. latifolius* had 38 rRNAs compared with 39 for *E. macropterus* and 37 for the other four species. Pseudogenisation was detected in *rps*16 (LSC), *ycf*1 (IRb), and *ndh*F (SSC) (Table 2), and *rps*16 was observed without non-coding genes (introns) in all the taxa studied. Pairwise determination of divergent regions was conducted using mVISTA, in which the six species were compared with *E. hamiltonianus* (KY926695) as a reference. Introns exhibited variation, whereas coding regions demonstrated a higher level of conservation (Figure 2).

### Nucleotide Diversity

**FIGURE 3:**
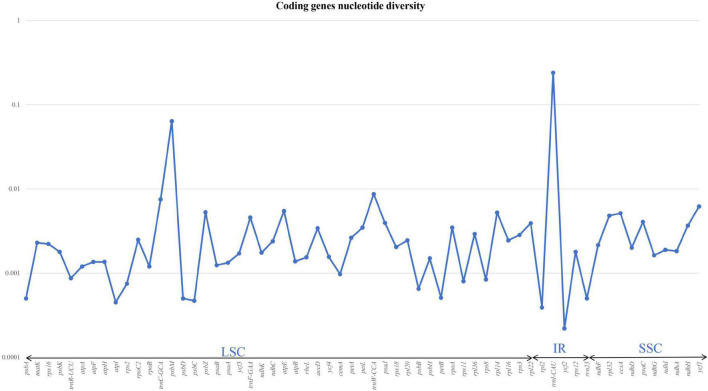
Graph illustrating the nucleotide variations in the coding genes of six sect. *Uniloculares* species. The graph does not include genes with zero variability.

Nucleotide diversity was analysed for both coding and non-coding regions of the six species. We aligned 114 genes from the LSC, SSC, and IR and calculated π values. Of these, 53 genes, including *psb*I, *rpo*C1, *rps*14, and *rps*4, exhibited no nucleotide diversity (π = 0). The other 61 genes exhibited nucleotide diversity; the highest overall value was recorded for *psb*M (π = 0.04); the highest value observed among tRNAs was for *trn*I-CAU (π = 0.2). The π-value was 0.006 for *ycf*1, whereas *psb*Z, *atp*E, and *rpl*14 had π-values of 0.005. We observed π-values of 0.003 in *rpo*A, *acc*D, *pet*l, *pas*J, and *rpl*22 (Figure 3). Detailed values are shown in Supplementary Table 2.

**FIGURE 4:**
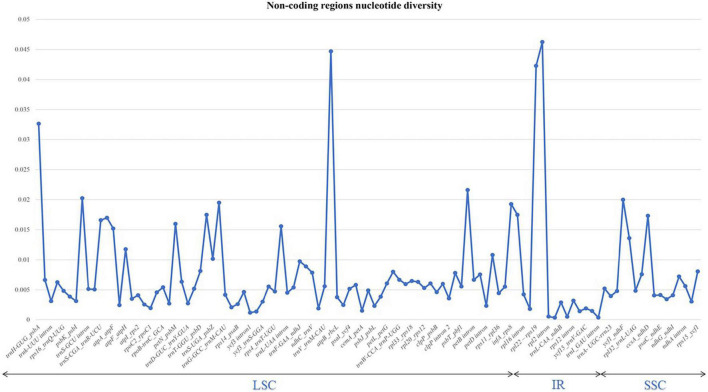
Graph illustrating the variations in nucleotide sequences in the non-coding regions (introns) of six sect. *Uniloculares* species. The graph does not include genes with zero variability.

The nucleotide diversity of non-coding regions was also analysed. We analysed 130 regions and introns between the genes. Of these, only 21 exhibited no diversity, including *rpo*C1_*rpo*B, *psa*B_*psa*A, and *ndh*J_*ndh*K. The highest π-value (0.04) observed was for *trn*M-CAU_*atp*E, *rpl*22_*rps*19, and *rps*19_*rpl*2. Other introns had π-values of 0.01 (Figure 4 and Supplementary Table 3).

### Phylogenetic Analysis

**FIGURE 5:**
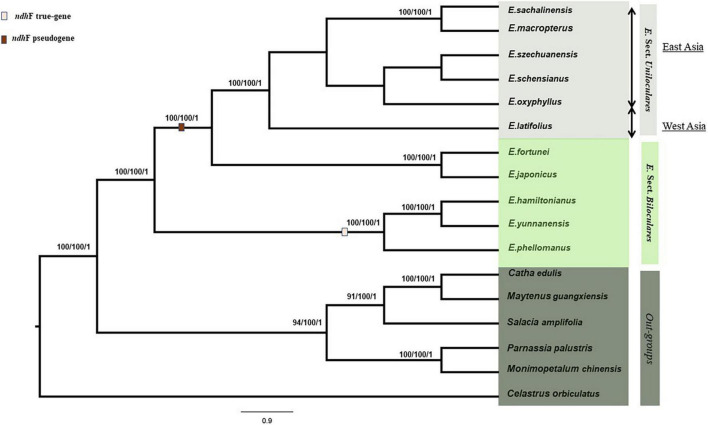
Maximum parsimony (MP), maximum likelihood (ML), and Bayesian inference (BI) were used to generate a phylogenetic tree based on whole chloroplast genomes of 11 *Euonymus* L. species (6 species of sect. *Uniloculares* and 5 of sect. *Biloculares*). Bootstrap values were 100, and the posterior probability values were 1. Values < 50 are ignored.

We constructed a phylogenetic tree based on 80 protein-coding genes in the cp genome using MP, ML, and BI. Only the ML tree is discussed further. A total of 11 *Euonymus* species were aligned with six Celastraceae species for which the whole cp genome data were available were used as outgroups, which includes *Catha edulis* (KT861471), *Celastrus orbiculatus* (MW316708), *Maytenus guangxiensis* (MN707924), *Monimopetalum chinense* (MK450440), *Parnassia palustris* (MH544205), and *Salacia amplifolia* (MK799641). We aligned 69,975 bps using MAFFT (Katoh et al., 2002). The six sect. *Uniloculares* species were grouped together in a clade with a bootstrap value of 100. This clade was monophyletic to sect. *Biloculares*, whereas the *Euonymus japonicus* and *Euonymus fortunei* clades were sister taxa to the sect. *Uniloculares* clade. Within the *Uniloculares* clade, *E. macropterus* and *E. sachalinensis* were sister taxa with 100% resolution. Sect. *Uniloculares* is closely related to *E. japonicus* and *E. fortunei*. *E. latifolius*, the representative taxon from western Asia, was located in a separate branch of sect. *Uniloculares* clade with 100% resolution. *E. japonicus* and *E. fortunei* were grouped in a clade and exhibited a close association, with a bootstrap value of 100%. *E. hamiltonianus*, *Euonymus yunnanensis*, and Euonymus *phellomanus* were grouped in a separate clade with 100% resolution within the sister group, whereas *E. hamiltonianus* and *E. yunnanensis* exhibited a 100% relationship to one another. Values < 50 are not shown in the tree. Within the *Euonymus* ingroup, *ndh*F was recorded as a true gene in *E. hamiltonianus*, *E. yunnanensis*, and *E. phellomanus*. In contrast, *ndh*F was a pseudogene in all members of sect. *Uniloculares* included in our analysis, along with *E. japonicus* and *E. fortunei*. The length of the gene was reduced above the clade. Pseudogenisation was also observed in *rps*16 (LSC) and *ycf*1 (IRb) in both the ingroups and outgroups (Figure 5).

### Boundaries Between the Inverted Repeat and Small Single Copy Regions

**FIGURE 6:**
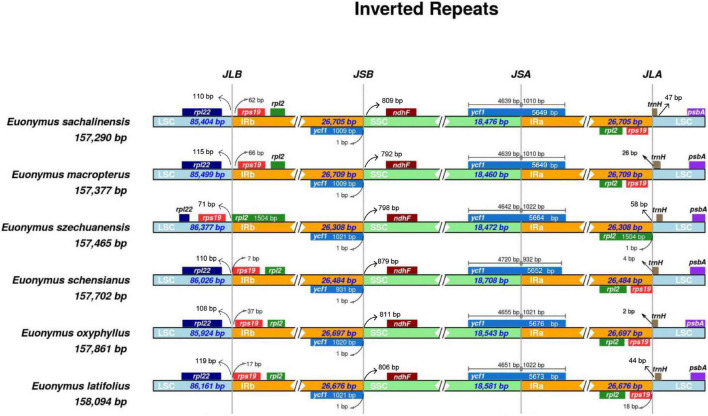
Large single copy (LSC), inverted repeat (IR), and small single copy (SSC) junction comparison among chloroplast genomes of six species of *Euonymus* sect. *Uniloculares.*

The IRs in the cp genomes of flowering plants differ only slightly among species (Goulding et al., 1996). We compared four plastid genomes along with two previously available genomes from sect. *Uniloculares* to investigate the expansion or contraction of the IR and SSC regions. An expansion of the IR junction is evident in Figure 6, where *rps*19 is present in both IRa and IRb. Except *E. szechuanensis*, the IR region in the other five species extended from 7 to 66 bp in IRb. The pseudogene *ycf*1 (IRb) was adjusted to the boundary between IRb and the SSC. Both the JLA and JLS appeared to be unstable, as *trn*H was observed to move toward the IR in all six species. Slight variations in *ycf*1, from 4,639 to 4,720 bp, were observed in the SSC. Pseudogenisation of *ndh*F was observed in all members of sect. *Uniloculares*, as its length was short compared with the true gene. As a pseudogene, *ndhF* is 792–879 bp from the junction JSB.

**FIGURE 7:**
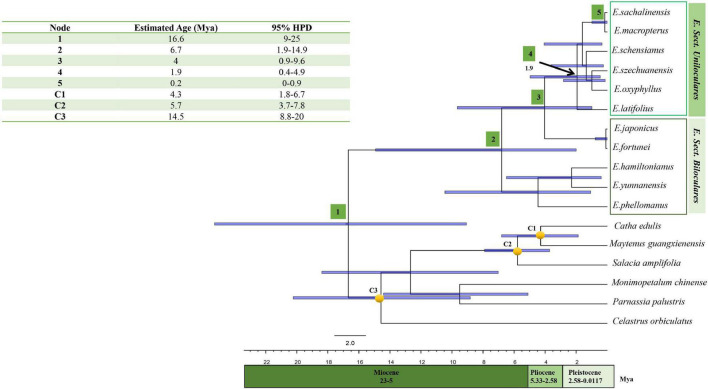
Chronogram depicting the divergence time estimated in BEAST using chloroplast coding sequences. Approximately 95% posterior probability is represented by the bar on each node. Nodes 1–5 are the nodes of interests. The calibration points utilised in the analysis were represented by nodes labelled as C1, C2, and C3.

### Time Divergence Estimation and Fossil Constraints

Our time-of-divergence estimate indicated that extant members of *Euonymus* diverged from other members of the Celastraceae approximately 16.6 Mya (Node 1; 95% HPD = 9–25 Mya), locating it within the Middle Miocene era. The crown age of the clade comprising *E. japonicus*, *E. fortunei*, and members of sect. *Uniloculares* was estimated to have diverged approximately 6.7 Mya (Node 2; 95% HPD = 1.9–14.4 Mya). The crown age of sect. *Uniloculares* was estimated to have occurred approximately 4.0 Mya (Node 3; 95% HPD = 0.9–9.6 Mya) during the Pliocene era. With respect to the divergence of *E. latifolius*, the western Asian representative of sect. *Uniloculares*, the estimated crown age of *E*. sect. *Uniloculares* was approximately 1.9 Mya (Node 4; 95% HPD = 0.4–4.9 Mya). Species in sect. *Uniloculares* diverged during the Pleistocene era. The clade was estimated to be young and to have diverged from ancestral taxa with two-celled anthers (*Biloculares*) (Figure 7).

### Vicariance, Disjunction, and Dispersal

**FIGURE 8:**
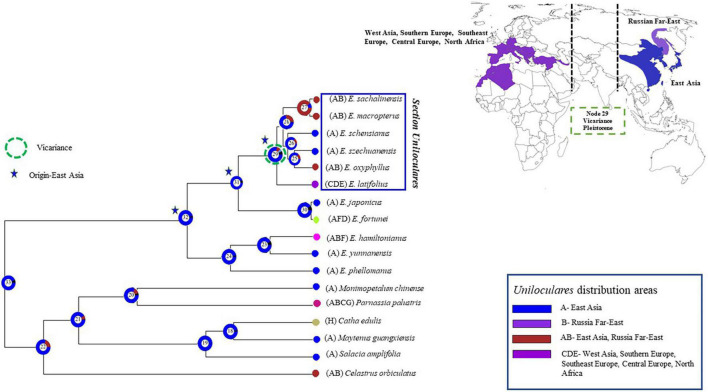
The Bayesian binary method (BBM) was used to analyse the biogeography of *Euonymus* sect. *Uniloculares*. The vicariance between East and West Asia is highlighted on the map to the right. The figure highlights four primary areas of interest. The Supplementary Table 4 contains the detail of each node’s events.

A biogeographical evaluation was conducted in RASP using the Bayesian binary method analysis. This assessment indicated that sect. *Uniloculares* originated in East Asia (Node 29, relative probability = 0.6). A vicariance event was also observed at Node 29. *Euonymus schenisanus* and *E. szechuanensis* are endemic to China (East Asia; A), whereas *E. macropeterus*, *E. sachalinensis*, and *E. oxyphyllus* also occur in the Russian Far East (East Asia–Russian Far East; AB). Only *E. latifolius* occurs in western Asia, south, southeast, and Central Europe, and North Africa (=CDE), making its distribution opposite to the East Asian sister clade (Figure 8).

**FIGURE 9:**
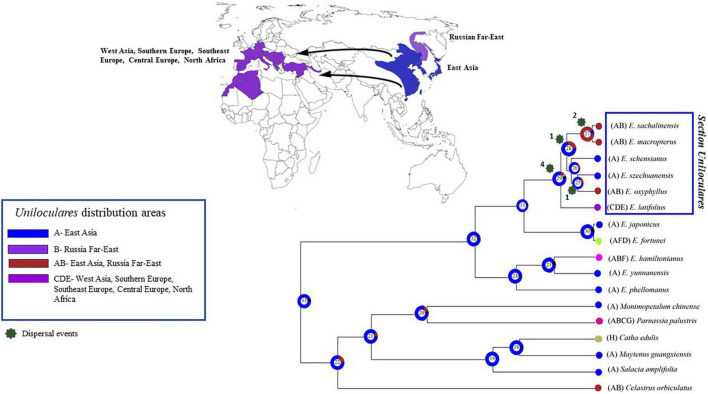
Figure illustrating the dispersal events. Number above star indicates the number of times of dispersal in the sect. *Uniloculares* clade. Node 29 is the main node of interest. Arrows on the map point the dispersal from East to West Asia. The Supplementary Table 4 contains the detail of each node’s events.

In the *Uniloculares* clade, four dispersal events occurred at Node 29, two at Nodes 27 and 25, and one at Node 28. No extinctions were observed (Figure 9).

## Discussion

### Pseudogenisation of *ndh*F and *rps*16

Pseudogenisation of the *ycf*1 gene in IRb is a common phenomenon in most cp genomes, but the loss of function in other coding genes is more intriguing. The first whole cp genome sequencing in *Euonymus* was the sequencing of *E. japonicus* according to the study by Choi and Park (2016), who observed pseudogenisation of *ndh*F. Pseudogenisation of *ndh*F has also been reported in the genomes of several other plant species, including *Saniculiphyllum* sp. (Saxifragaceae) (Folk et al., 2020), *Zoysia* sp., *Sporobolus* sp. (Poaceae) (Cheon et al., 2021), and some members of the Orchidaceae (Zavala-Páez et al., 2020). Intron loss in *rps*16 has been confirmed in eight members of the Celastraceae and was successfully detected by primers designed to identify such losses (Gu et al., 2018). Pseudogenenisation of *rps*16 has been reported in *Gentiana straminea* (Ni et al., 2016), *Gentiana crassicaulis*, and *Gentiana robusta* (Ni et al., 2017), as well as in *Hepatica* sp. (Li et al., 2021), *Populus* sp., *Veratrum* sp., non-parasitic members of the Fabaceae, and members of the Orchidaceae (Nagano et al., 1991; Saski et al., 2005; Tuskan et al., 2006; Ni et al., 2016).

### Phylogeny

*Euonymus* is difficult to identify at the species level because the flowers are not showy and shed shortly after blooming, complicating the task of identifying specimens based on reproductive structures (Du et al., 2016). As a result, species identifications are disputed, and taxonomists are currently attempting to resolve the debate using genetic data. Wang et al. (2020) constructed phylogenetic trees based on whole cp genomes, in which *E. schensianus* and *E. szechuanensis* were grouped together in a clade with 100% resolution. *E. japonicus* was grouped in a subclade with the latter, closely approximating our results. According to the study by Simmons et al. (2012), who analysed *mat*K, *trn*L-F, ITS, and 26S rDNA, sect. *Uniloculares* (=subgenus *Kalonymus*) was placed in a subclade with sect. *Illicifolia*, which includes *E. japonicus* and *E. fortunei*.

### Time of Divergence and Vicariance

We propose that the diversification of the sect. *Uniloculares* clade occurred in East Asia in the late Pliocene, approximately 4 Mya (Node 3) (Figure 7). In their study, Wang et al. (2019) confirmed that several climatic changes occurred in East Asia throughout the Pliocene epoch, including an unanticipated change in monsoon cycles approximately 4.2 Mya due to high-latitude cooling.

The mid-Pliocene warm period occurs between 3.264 and 3.025 Mya, during which global temperatures are considered warmer than current temperatures, whereas paleogeography appears to be modern. These warm ages are assumed to share many parallels with the global warming and carbon dioxide levels (Meng and Kubatko, 2009) observed throughout the past century, with global temperature estimates ranging from 1.8 to 3.6°C (Dowsett et al., 2010; Haywood et al., 2013; Zhang et al., 2013).

The evolution and diversification of the Asian flora can be traced back to the formation of the Gobi Desert, which is situated in an arid region of Asia. This change is considered to have occurred 2.6 Mya and may be attributable to changes in geography and climate. Other potential causal factors include geological deformation and changes in the Asian monsoon due to global warming (Lu et al., 2019). The desert could potentially limit gene flow. Global climatic oscillations occurred approximately 2.0 Mya, during the Pleistocene epoch, causing environmental changes that led to the development and migration of numerous species in the Northern Hemisphere (Hewitt, 2000; Hewitt, 2004; Qiu et al., 2011).

The diversification of species in sect. *Uniloculares* is estimated to have occurred between 0.9 and 1.9 Mya and appears to have originated in East Asia. *E. latifolius*, the western Asian representative, diverged approximately 1.9 Mya, indicating that the clade diverged recently. Species that diverged around the same time as the East Asian clade of sect. *Uniloculares* due to phylogeographic segregation in East Asia include *Platycrater arguta* (Hydrangeaceae; 0.89 Mya) (Qiu et al., 2009b), *Kirengeshoma koreana* and *Kirengeshoma palmata* (Hydrangeaceae; 0.45 and 0.16 Mya, respectively) (Qiu et al., 2009c), and *Dysosma versipellis* (Berberidaceae; 0.4 Mya) (Qiu et al., 2009a). According to the study by Wadia (1955), available evidence implies that desertification occurred after glacial events. There were seven or eight well-established oscillations during the Pleistocene ice age. It is thought that such fluctuations, e.g., from cold to warm temperatures, occurred approximately 10,000–15,000 years ago.

In recent years, phylogeographic studies have investigated the connection between the evolution of organisms and geological uplift. Mountainous uplifts result in the formation of geographical barriers, leading to habitat fragmentation, loss of dispersal corridors, and restricted gene flow (Luo et al., 2016). Due to previous climatic changes, eastern and western Asia became colonised by numerous trees species (Wolfe, 1975; Qiu et al., 2011; Gratzfeld, 2013). Song et al. (2020) discussed disjunction and vicariance between eastern and western Asia using *Petrocarya* spp. (Juglandaceae) as a model species and posited the formation of the Gobi Desert as a factor in the disjunction between eastern and western Asia. Woody taxa such as *Fagus* (Fagaceae), *Acer* (Sapindaceae), *Buxus* (Buxaceae), *Sorbus* (Rosaceae), and *Parrotia* (Hamamelidaceae) are reported to have similar distribution patterns in eastern and western Asia (Ickert-Bond et al., 2005; Li and Tredici, 2008; Jia and Bartish, 2018; Kozlowski et al., 2018).

Our study provides an evolutionary framework for evaluating the biogeographic history of *Euonymus* sect. *Uniloculares*. The monophyly of sect. *Uniloculares* was supported based on analyses of whole cp genomes. We posit that East Asia is the centre of origin of sect. *Uniloculares*. Temperature fluctuations, desertification, and changes in rainfall cycles may have played a role in the disjunction by disrupting gene flow between regions. Scientific exploration and experimentation are ongoing, and more studies should be conducted on the phylogenomics, evolution, demographic history, and biogeography of *Euonymus*, as many questions about the genus remain unanswered.

## Data Availability Statement

The datasets presented in this study can be found in online repositories. The names of the repository/repositories and accession number(s) can be found below: National Center for Biotechnology Information (NCBI) BioProject database under accession numbers OL770076-OL770079.

## Author Contributions

All authors listed have made a substantial, direct, and intellectual contribution to the work, and approved it for publication.

## Conflict of Interest

The authors declare that the research was conducted in the absence of any commercial or financial relationships that could be construed as a potential conflict of interest.

## Publisher’s Note

All claims expressed in this article are solely those of the authors and do not necessarily represent those of their affiliated organizations, or those of the publisher, the editors and the reviewers. Any product that may be evaluated in this article, or claim that may be made by its manufacturer, is not guaranteed or endorsed by the publisher.

## Abbreviations

BI: Bayesian inference
BBM: Bayesian binary method
Bp: base pairs
Cp: chloroplast
HPD: highest posterior density
IR: inverted repeat
LSC: large single copy
MCMC: Markov Chain Monte Carlo
Mya: million years ago
ML: maximum likelihood
MP: maximum parsimony
NCBI: National Centre for Biotechnology Information
sect.: section
st. dev.: standard deviation
sp.: species
SSC: small single copy.
