# Supplementary material for: Disjunction and Vicariance Between East and West Asia: A Case Study on Euonymus sect. Uniloculares Based on Plastid Genome Analysis
Source: Front Plant Sci. 2022 Mar 11;13:825209. doi: 10.3389/fpls.2022.825209 (PMC8963480; doi:10.3389/fpls.2022.825209)
Supplement: Supplementary file 1 [file Table_1.docx]

**[S-1] Accession Numbers of *Celastraceae* members used in the study**

| **S.No** | **Bot. Name** | **NCBI Accession Number** |
| --- | --- | --- |
|  | *E. oxyphyllus* Miq. | OL770078 |
|  | *E. macropterus* Rupr. | OL770077 |
|  | *E. latifolius* (L.) Mill. | OL770076 |
|  | *E. sachalinensis* (F.Schmidth) Maxim. | OL770079 |
|  | *E.schensianus* Maxim. | KY511610 |
|  | *E.szechuanensis* C.H Wang | MH853828 |
|  | *E. phellomanus* Loes. | MW288092.1 |
|  | *E.japonicus* Thunb. | KP189362 |
|  | *E. fortunei*(Turcz.) Hand.-Mazz. | MW288090.1 |
|  | *E.hamiltonianus* Wall. | KY926695 |
|  | *E. yunnanensis* Franch. | MW770452 |
|  | *Monimopetalum chinense* Rehder. | MK450440 |
|  | *Maytenus guangxiensis* C.Y. Cheng & W.L.Sha | MN707924 |
|  | *Salacia amplifolia* Merr. ex Chun & F.C.How | MK799641 |
|  | *Catha edulis* (Vahl) Endl. | KT861471 |
|  | *Parnassia palustris* L. | MK541646.1 |
|  | *Celastrus orbiculatus* Thunb | MW316708 |
