# Supplementary material for: Disjunction and Vicariance Between East and West Asia: A Case Study on Euonymus sect. Uniloculares Based on Plastid Genome Analysis
Source: Front Plant Sci. 2022 Mar 11;13:825209. doi: 10.3389/fpls.2022.825209 (PMC8963480; doi:10.3389/fpls.2022.825209)
Supplement: Supplementary file 4 [file Table_4.DOCX]

**[S-4] Detail information of RASP analysis**

| **Node** | **Event Matrix** | | | | |
| --- | --- | --- | --- | --- | --- |
|  | **Dispersal** | **Vicariance** | **Extinction** | **Probability** | **Event Route** |
| NODE18 | 2 | 1 | 0 | 0.8306 | A->AH->H\|A |
| NODE19 | 0 | 0 | 0 | 0.8051 | A->A^A->A\|A |
| NODE20 | 3 | 0 | 0 | 0.9925 | A->A^A->ABCG^A->A\|ABCG |
| NODE21 | 0 | 0 | 0 | 0.8887 | A->A^A->A\|A |
| NODE22 | 1 | 0 | 0 | 0.7653 | A->A^A->AB^A->AB\|A |
| NODE23 | 2 | 0 | 0 | 0.9449 | A->A^A->ABF^A->ABF\|A |
| NODE24 | 0 | 0 | 0 | 0.9317 | A->A^A->A\|A |
| NODE25 | 1 | 0 | 0 | 0.8508 | A->A^A->AB^A->A\|AB |
| NODE26 | 0 | 0 | 0 | 0.7173 | A->A^A->A\|A |
| NODE27 | 2 | 0 | 0 | 0.9031 | AB->AB^A^B->AB\|AB |
| NODE28 | 1 | 0 | 0 | 0.5627 | A->A^A->AB^A->AB\|A |
| NODE29 | 4 | 1 | 0 | 0.6024 | A->ACDE->CDE\|A |
| NODE30 | 2 | 0 | 0 | 0.9259 | A->A^A->ADF^A->A\|AFD |
| NODE31 | 0 | 0 | 0 | 0.7107 | A->A^A->A\|A |
| NODE32 | 0 | 0 | 0 | 0.9081 | A->A^A->A\|A |
| NODE33 | 0 | 0 | 0 | 0.7783 | A->A^A->A\|A |

Dispersal Between Areas:

A->B:5

A->C:2

A->D:2

A->E:1

A->F:2

A->G:1

A->H:1

Speciation Within Areas:

A:14

B:1

Dispersal Table:

|  | from | to | within |
| --- | --- | --- | --- |
| A | 14.00 | 0.00 | 14 |
| B | 0.00 | 5.00 | 1 |
| C | 0.00 | 2.00 | 0 |
| D | 0.00 | 2.00 | 0 |
| E | 0.00 | 1.00 | 0 |
| F | 0.00 | 2.00 | 0 |
| G | 0.00 | 1.00 | 0 |
| H | 0.00 | 1.00 | 0 |

Global Cost:

Global Dispersal: 18

Global Vicariance: 2

Global Extinction: 0
